# The impact of antifibrotic use on long-term clinical outcomes in the pulmonary fibrosis foundation registry

**DOI:** 10.1186/s12931-024-02883-2

**Published:** 2024-06-21

**Authors:** Cathryn T. Lee, Wei Hao, Cindy A. Burg, Jennie Best, Giselle E. Kolenic, Mary E. Strek

**Affiliations:** 1 Section of Pulmonary and Critical Care Medicine, Department of Medicine, University of Chicago, 5841 S. Maryland Ave, Chicago, IL 60637 USA; 2 Department of Biostatistics, Statistical Analysis of Biomedical and Educational Research Group (SABER), University of Michigan, Ann Arbor, MI USA; 3 U.S. Medical Affairs, Genentech, Inc., South San Francisco, CA USA

**Keywords:** Interstitial lung disease, Pirfenidone, Nintedanib, Antifibrotic

## Abstract

**Background:**

Idiopathic pulmonary fibrosis (IPF) is a devastating interstitial lung disease (ILD) with a high mortality rate. The antifibrotic medications pirfenidone and nintedanib have been in use since 2014 for this disorder and are associated with improved rate of lung function decline. Less is known about their long-term outcomes outside of the clinical trial context.

**Methods:**

The Pulmonary Fibrosis Foundation Patient Registry was used for this study. Patients with an IPF diagnosis made within a year of enrollment were included. The treated group was defined as patients receiving either pirfenidone or nintedanib for at least 180 days. The untreated group did not have any record of antifibrotic use. Demographic data, comorbidities, serial lung function, hospitalization, and vital status data were collected from the registry database. The primary outcomes were transplant-free survival, time to first respiratory hospitalization, and time to 10% absolute FVC decline. Time-to-event analyses were performed utilizing Cox proportional hazards models and the log-rank test. Model covariates included age, gender, smoking history, baseline lung function, comorbidities, and oxygen use.

**Results:**

The registry contained 1212 patients with IPF; ultimately 288 patients met inclusion criteria for the treated group, and 101 patients were designated as untreated. Patients treated with antifibrotics were significantly younger (69.8 vs. 72.6 years, *p* = 0.008) and less likely to have smoked (61.1% ever smokers vs. 72.3% never smokers, *p* = 0.04). No significant differences were seen in race, gender, comorbidities, or baseline pulmonary function between groups. The primary outcome of transplant-free survival was not significantly different between the two groups (adjusted HR 0.799, 95% CI 0.534–1.197, *p* = 0.28). Time to respiratory hospitalization was significantly shorter in the treated group (adjusted HR 2.12, 95% CI 1.05–4.30, *p* = 0.04). No significant difference in time to pulmonary function decline was seen between groups.

**Conclusions:**

This multicenter study demonstrated 63% of newly diagnosed IPF patients had continuous antifibrotic usage. Antifibrotics were not associated with improved transplant-free survival or pulmonary function change but was associated with an increased hazard of respiratory hospitalization. Future studies should further investigate the role of antifibrotic therapy in clinically important outcomes in real-world patients with IPF and other progressive ILDs.

**Supplementary Information:**

The online version contains supplementary material available at 10.1186/s12931-024-02883-2.

## Background

Idiopathic pulmonary fibrosis (IPF) is a progressive and fatal pulmonary condition with high morbidity and median survival of 3–5 years [1]. Crucially, the antifibrotic agents pirfenidone and nintedanib were approved by the Food and Drug Administration (FDA) in 2014 for the treatment of IPF. Pirfenidone’s mechanism of action is incompletely understood, but it contributes to the suppression of a variety of inflammatory and fibrotic pathways [2]. Nintedanib, a tyrosine kinase inhibitor, inhibits pro-fibrotic growth factor receptors and extracellular matrix deposition [3].

While not a cure, antifibrotics have demonstrated effectiveness in randomized controlled trials and slow the rate of decline of lung function in patients with IPF [4, 5]. These studies demonstrated a clear benefit in forced vital capacity (FVC) change at one year after therapy initiation. Less established, however, are the effects of antifibrotics on longer-term outcomes, including transplant-free mortality. While some retrospective studies have shown increased survival in IPF patients receiving antifibrotics, others have failed to find a significant association [6–8]. This variability could be due to differences in cohort selection, duration of therapy, and approach to minimizing confounding between treated and untreated groups. Additionally, the characteristics of patients, including demography, comorbidities, symptoms, and geography, that are associated with antifibrotic use outside of the controlled trial setting are less understood.

This study utilized the Pulmonary Fibrosis Foundation Patient Registry (PFF-PR) to examine the characteristics and outcomes of patients treated with antifibrotic agents in a real-world multicenter patient cohort. We hypothesized that patients on antifibrotic therapy would be younger, have fewer comorbidities, decreased numbers of respiratory hospitalization, and improved survival compared to patients not receiving antifibrotic therapy.

## Methods

### Cohort description

This retrospective cohort study utilized the Pulmonary Fibrosis Foundation Patient Registry (PFF-PR), a registry of over 2000 patients seeing physicians from the PFF Care Center Network, the largest collection of ILD clinics in the United States (US) [9]. Data collection began in 2016, two years after the FDA approved pirfenidone and nintedanib for use in the US. All patients provided written informed consent, and IRB approval was granted via each individual participating center. Demographic data, comorbidities, medication information, serial lung function, radiologic data, and vital status information are collected on participants regularly. Patients also completed the Fatigue Severity Scale, Leicester Cough Questionnaire, Rand SF-6D questionnaire, and UCSD shortness of breath questionnaires both at enrollment and at subsequent visits.

Follow-up data was collected for each individual patient by their respective PFF Care Center via data abstraction forms completed every six months [9]. These forms contained information regarding any office visits, test results, or hospitalization in the interim, including updates to medications, pulmonary function testing, diagnoses, oxygen use, hospitalizations, referrals to palliative care, transplantation, and death. Patients who have not returned within twelve months of enrollment into the study were contacted by the individual PFF Care Center staff in attempts to complete assessments.

### Patient selection, primary and secondary outcomes

IPF patients enrolled in the registry within one year of diagnosis were included in this study. This restriction by diagnosis time was made in an attempt to limit variability of disease severity at the time of study entry. Patients were defined as “treated” if they were recorded as ever taking pirfenidone or nintedanib for a duration of at least 180 days continuously after consent into the registry. We chose 180 days as the continuous treatment time threshold to ensure that drug was taken for long enough to be considered effective. Patients that received pirfenidone and nintedanib for greater than 0 but less than 180 days were included in a sensitivity analysis. Patients were defined as “untreated” if they were recorded as not being on pirfenidone or nintedanib for the duration of follow-up. The primary outcomes studied were transplant-free survival, respiratory hospitalization, and time to 10% absolute FVC decline. Time zero for all outcomes was at study enrollment for both treated and untreated groups. Event time was restricted to 2000 days since enrollment. Secondary outcomes studied were survival (with transplant a censoring event) and time to 5% absolute FVC decline. In the treatment group, the prespecified 5% and 10% thresholds for FVC decline were not noted as an event if they occurred prior to 180 days of treatment.

### Statistical analysis

T-tests and Chi squared tests were performed to compare treated and untreated groups for continuous and categorical variables, respectively. Fisher’s exact test was used for categorical variables when counts were less than 5. Time-to-event analyses compared treated and untreated groups for the primary and secondary outcomes utilizing Cox proportional hazards models and the log-rank test. Both unadjusted analyses as well as those adjusted for age, gender, smoking history, baseline lung function, select comorbidities known to affect survival and hospitalization rates (coronary artery disease (CAD), chronic obstructive pulmonary disease (COPD), and pulmonary hypertension), and oxygen use were performed [10, 11]. Kaplan-Meier curves were generated to visualize time to primary and secondary outcomes by antifibrotic use. A Poisson regression model was employed for the outcome of frequency of respiratory hospitalization. All statistical analyses were performed using SAS version 9.4 (Cary, NC, USA).

## Results

**Fig. 1:**
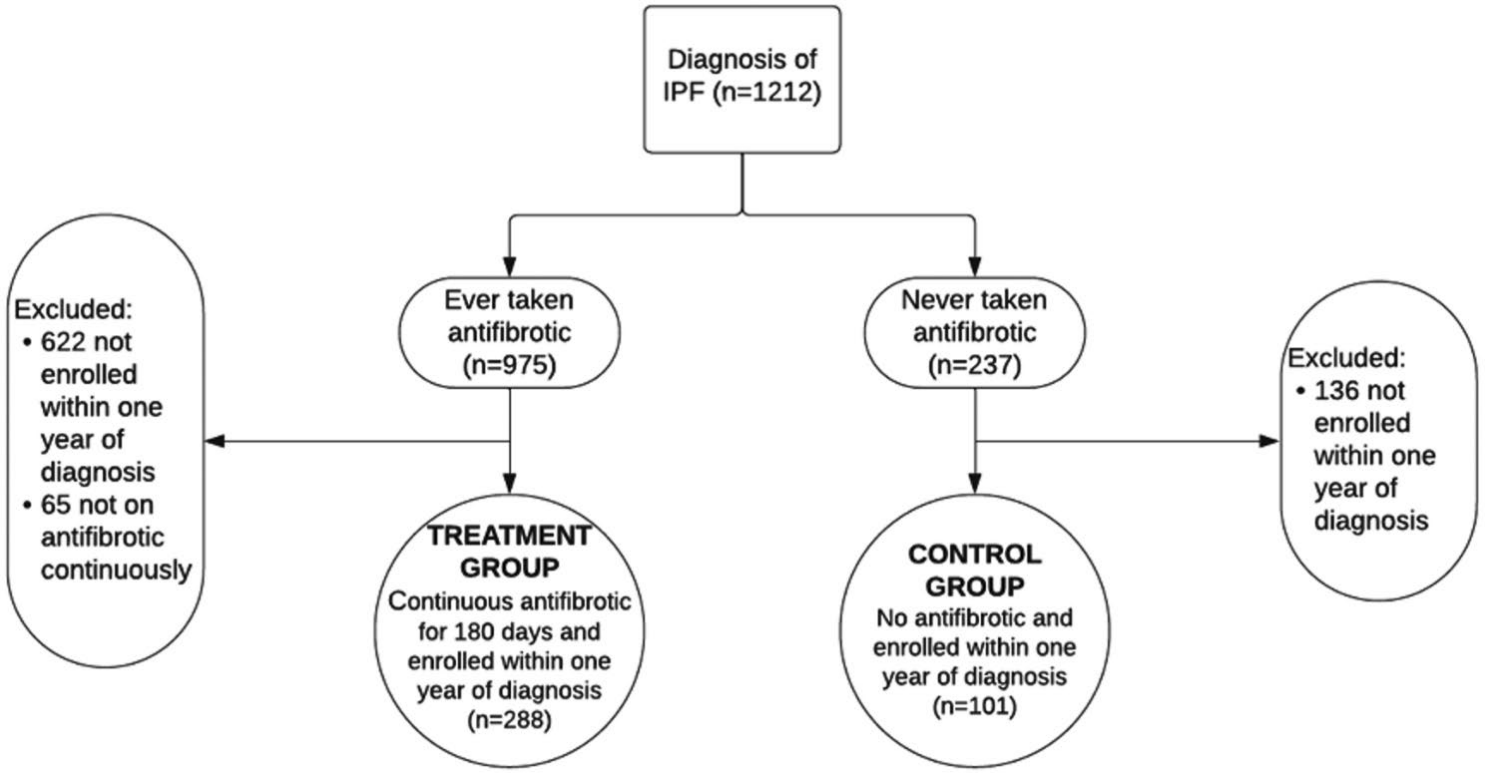
Patient classification

Twelve hundred twelve patients in the PFF Registry had a diagnosis of IPF. The mean time between diagnosis of IPF and enrollment into the registry was 910 days. Three hundred and eighty-nine patients met the inclusion criteria of (1) IPF diagnosis within one year of consent and (2) either experienced 180 days or more of continuous antifibrotic exposure (treatment group, 288 patients) or 0 days of antifibrotic exposure (untreated group, 101 patients); this comprised our study cohort. Those excluded were 622 patients on antifibrotics but not enrolled within a year of diagnosis, 65 patients enrolled within a year of diagnosis but not on antifibrotics continuously, and 136 patients who were never on antifibrotics and were not consented within one year of diagnosis (Fig. 1). In the treatment group, the mean duration of antifibrotic use was 1113 days (range: 186–2106 days).

The mean age of the cohort was 71, 81% were male, and 96% were White (Table 1). Patients treated with antifibrotics were significantly younger (69.8 vs. 72.6 years, *p* = 0.008) and less likely to have smoked (61.1% ever smokers vs. 72.3% never smokers, *p* = 0.04) than their untreated counterparts. Additionally, patients treated with antifibrotics had a statistically significantly longer time since diagnosis (mean 146 days vs. 110 days in untreated group), but this difference was small in magnitude. No significant differences were seen in race, ethnicity, BMI, comorbidities, insurance status, or geography between treated and untreated patients. No significant differences in measures of baseline pulmonary function were observed.

**Table 1:** Patient characteristics

	Total Cohort (*n* = 389)	Antifibrotic (*n* = 288)	No Antifibrotic (*n* = 101)	*p*-value*
Age, mean (SD), years	70.6(7.9) (*n* = 389)	69.8(7.2) (*n* = 288)	72.6(9.5) (*n* = 101)	0.008
Male, n (%)	313(80.5) (*n* = 389)	230(79.9) (*n* = 288)	83(82.2) (*n* = 101)	0.61
Race				
White, n (%)	359(95.7)	269(96.4)	90(93.8)	0.35
Black, n (%)	4(1.1)	3(1.1)	1(1.0)	
Asian, n (%)	12(3.2)	7(2.5)	5(5.2)	
	(*n* = 375)	(*n* = 279)	(*n* = 96)	
Hispanic, n (%)	18(4.8)	13(4.7)	5(5.3)	0.79
	(*n* = 372)	(*n* = 277)	(*n* = 95)	
Body mass index, mean (SD)	29.5(5.2)	29.7(5.1)	29.2(5.6)	0.48
	(*n* = 358)	(*n* = 267)	(*n* = 91)	
Days since diagnosis, mean (SD)	136.7(118.4)	146.1(118.3)	109.9(115.1)	0.008
	(*n* = 389)	(*n* = 288)	(*n* = 101)	
Region				0.71
West, n (%)	85(21.9)	65(22.6)	20(19.8)	
Midwest, n (%)	67(17.2)	46(16.0)	21(20.8)	
South, n (%)	161(41.4)	121(42.0)	40(39.6)	
Northeast, n (%)	76(19.5)	56(19.4)	20(19.8)	
	(*n* = 389)	(*n* = 288)	(*n* = 101)	
Insurance				0.77
Private, n (%)	208(53.5)	155(53.8)	53(52.5)	
Medicare, n (%)	157(40.4)	114(39.6)	43(42.6)	
Others, n (%)	24(6.2)	19(6.6)	5(5.0)	
	(*n* = 389)	(*n* = 288)	(*n* = 101)	
History of smoking tobacco, n (%)	249(64.0)	176(61.1)	73(72.3)	0.04
	(*n* = 389)	(*n* = 288)	(*n* = 101)	
No. of medical comorbidities, mean (SD)	2.0(1.5)	2.0(1.4)	2.1(1.8)	0.60
	(*n* = 389)	(*n* = 288)	(*n* = 101)	
Medical comorbidities				
GERD, n (%)	44(11.3)	29(10.1)	15(14.9)	0.19
OSA, n (%)	88(22.6)	64(22.2)	24(23.8)	0.75
Arrhythmia, n (%)	46(11.8)	33(11.5)	13(12.9)	0.71
CAD, n (%)	93(23.9)	68(23.6)	25(24.8)	0.82
CHF, n (%)	16(4.1)	11(3.8)	5(5.0)	0.57
COPD, n (%)	31(8.0)	21(7.3)	10(9.9)	0.40
Cancer, n (%)	65(16.7)	43(14.9)	22(21.8)	0.11
Depression, n (%)	50(12.9)	37(12.9)	13(12.9)	1.00
Diabetes, n (%)	72(18.5)	58(20.1)	14(13.9)	0.16
Cirrhosis, n (%)	2(0.5)	1(0.4)	1(1.0)	0.45
Obesity, n (%)	68(17.5)	52(18.1)	16(15.8)	0.61
PAH, n (%)	11(2.8)	7(2.4)	4(4.0)	0.49
	(*n* = 389)	(*n* = 288)	(*n* = 101)	
Family history of ILD				0.81
Yes, n (%)	65(16.7)	50(17.4)	15(14.9)	
No, n (%)	278(71.5)	205(71.2)	73(72.3)	
Unknown, n (%)	46(11.8)	33(11.5)	13(12.9)	
	(*n* = 389)	(*n* = 288)	(*n* = 101)	

SD = standard deviation; GERD = gastro-esophageal reflux disease, OSA = obstructive sleep apnea, CAD = coronary artery disease, CHF = congestive heart failure, COPD = chronic obstructive pulmonary disease, PAH = pulmonary hypertension, ILD = interstitial lung disease

In unadjusted analyses, untreated patients had significantly more baseline fatigue and shortness of breath as demonstrated by their mean Fatigue Severity Scale and UCSD Shortness of Breath scores (Table 2). No overall difference in mortality was detected between groups, and the proportion of patients who had a respiratory hospitalization was significantly higher in the treated compared to the untreated group.

**Table 2:** Pulmonary function, oxygen use, and patient-reported outcomes by antifibrotic use

	Total Cohort (*n* = 389)	Antifibrotic (*n* = 288)	No Antifibrotic (*n* = 101)	*p*-value
FVC % predicted, baseline, mean (SD)	70.6(16.3) (*n* = 314)	70.6(15.5) (*n* = 235)	70.6(18.7) (*n* = 79)	1.00
DLCO uncorrected % predicted, baseline, mean (SD)	45.3(14.6) (*n* = 278)	45.1(14.1) (*n* = 211)	45.7(16.2) (*n* = 67)	0.78
Gender-Age-Physiology score	4.1(1.3) (*n* = 278)	4.1(1.3) (*n* = 211)	4.2(1.3) (*n* = 67)	0.47
Supplemental oxygen use, n (%)	141(36.8) (*n* = 383)	108(38.0) (*n* = 284)	33(33.3) (*n* = 99)	0.40
Fatigue Severity Scale score, mean (SD)	3.9(1.8) (*n* = 375)	3.7(1.7) (*n* = 279)	4.3(1.9) (*n* = 96)	0.01
Leicester Cough Questionnaire score, mean (SD)	16.9(3.5) (*n* = 373)	17.1(3.3) (*n* = 278)	16.3(3.9) (*n* = 95)	0.07
Rand SF-6D Health-related Quality of Life score, mean (SD)	0.7(0.1) (*n* = 374)	0.7(0.1) (*n* = 278)	0.7(0.1) (*n* = 96)	0.07
UCSD Shortness of Breath-score, mean (SD)	33.3(24.1) (*n* = 370)	31.6(22.4) (*n* = 276)	38.4(28.1) (*n* = 94)	0.03
Death (all-cause)	118(30.3) (*n* = 389)	83(28.8) (*n* = 288)	35(34.7) (*n* = 101)	0.27
Respiratory hospital visit	102 (26.2) (*n* = 389)	90(31.3) (*n* = 288)	12(11.9) (*n* = 101)	0.0001

FVC = forced vital capacity; DLCO = diffusing capacity of carbon monoxide; UCSD = University of California San Diego

**Fig. 2:**
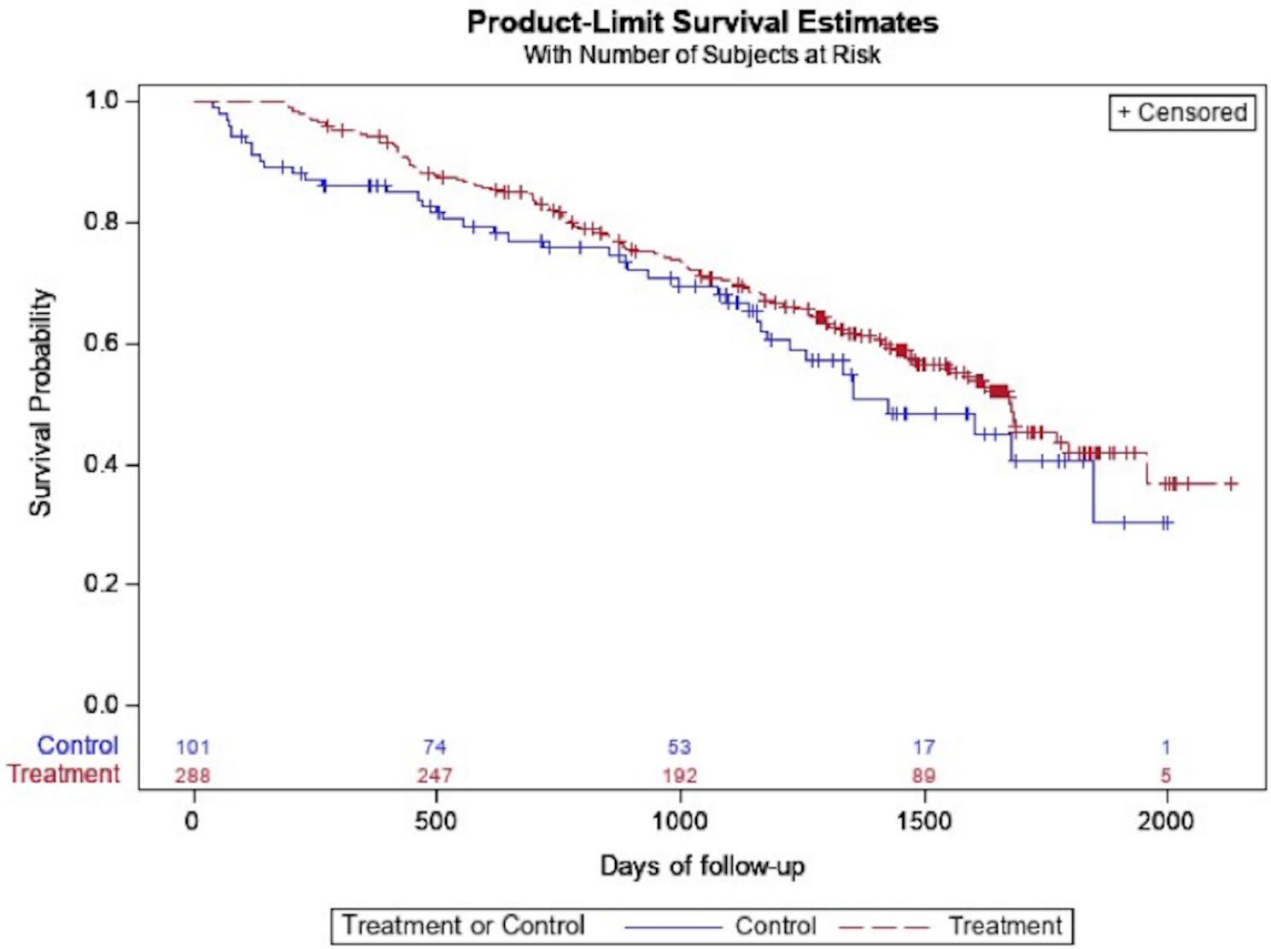
Unadjusted time to composite outcome of death or lung transplant, stratified by antifibrotic use (treatment = antifibrotic, control = no antifibrotic)

**Fig. 3:**
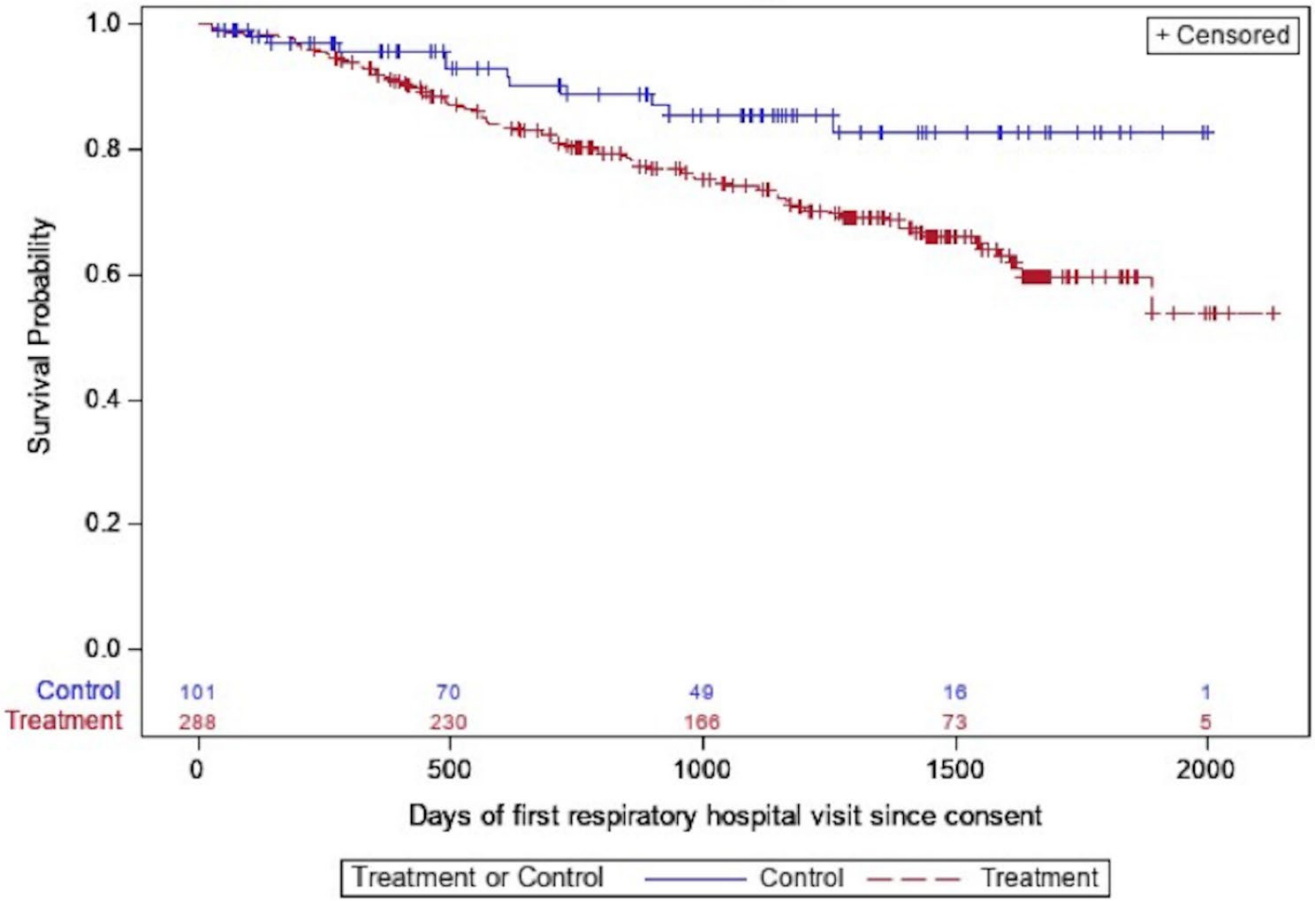
Unadjusted time to respiratory hospitalization, stratified by antifibrotic use (treatment = antifibrotic, control = no antifibrotic)

**Fig. 4:**
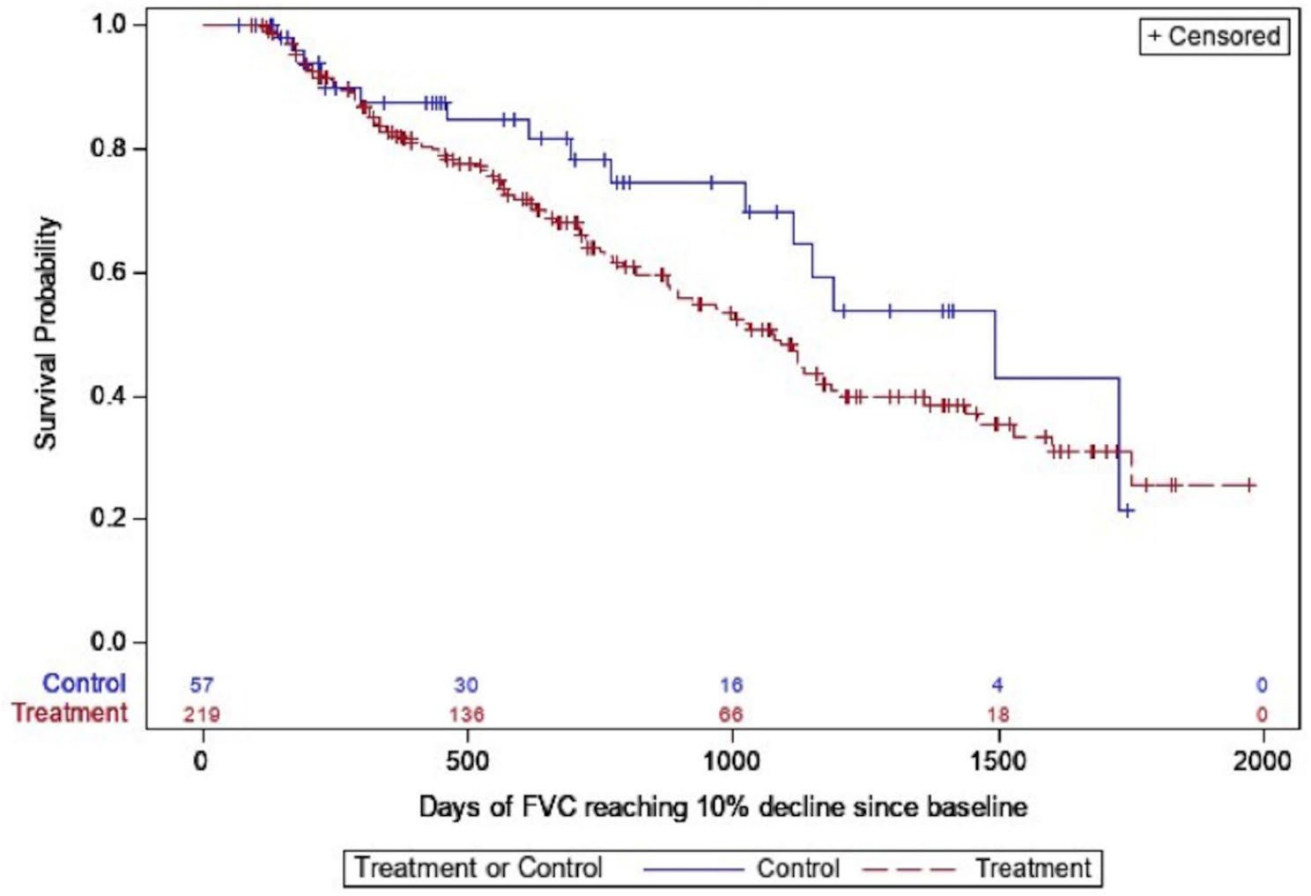
Unadjusted time to absolute FVC decline of 10%, stratified by antifibrotic use (treatment = antifibrotic, control = no antifibrotic)

**Fig. 5:**
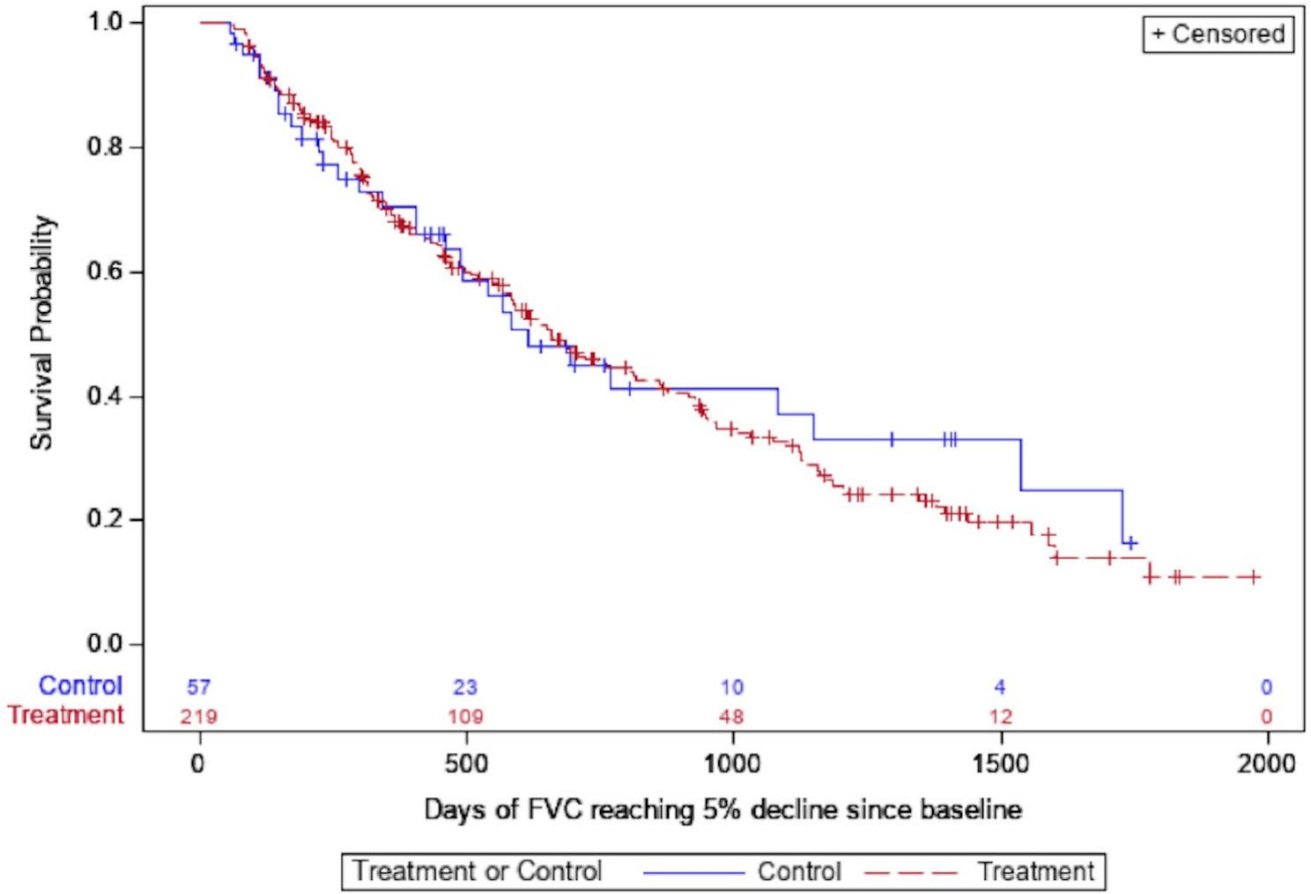
Unadjusted time to absolute FVC decline of 5%, stratified by antifibrotic use (treatment = antifibrotic, control = no antifibrotic)

The restricted mean transplant free survival time was 1567 days in the entire cohort; 1614 days in those treated with antifibrotics compared with 1422 days in the untreated group. While antifibrotic usage was associated with a significant reduction in death in the unadjusted analysis, the primary outcome of time to composite outcome of death or lung transplant was not significantly different between the two groups (Table 3; Fig. 2). The hazard of respiratory hospitalization was significantly higher in the treated group in both unadjusted and adjusted analyses (HR 2.12, 95% CI 1.05–4.30, *p* = 0.04, Table 3; Fig. 3). Patients on antifibrotics had an incident rate ratio of 2.60 (95% CI, 1.42–4.76, *p* = 0.002) of respiratory hospitalizations compared to patients not on antifibrotics, after multivariable adjustment. No significant differences in time to pulmonary function decline, regardless of FVC threshold used, was seen between groups (Table 3; Figs. 4 and 5). Similar effects were seen in a sensitivity analysis in which the same methods were used after removing patients in the study for less than six months total.

**Table 3:** Hazard ratios of death, lung transplant, and hospitalization by antifibrotic use

Outcome	Unadjusted HR*		Adjusted HR**	
	Hazard ratio (95% CI)	*P*-value	Hazard ratio (95% CI)	*P*-value
Death/Transplant	0.788 (0.557, 1.115)	0.18	0.799 (0.534, 1.197)	0.28
Death	0.621 (0.418, 0.924)	0.02	0.724 (0.454, 1.154)	0.17
Respiratory hospitalization	2.180 (1.193,3.984)	0.01	2.122 (1.047,4.301)	0.04
FVC 10% decline^$^	1.420 (0.848,2.376)	0.18	1.385 (0.822,2.335)	0.22
FVC 5% decline^%^	1.079 (0.727,1.600)	0.71	1.012 (0.676,1.516)	0.95

*Hazard ratio-hazard of outcome in patients treated with antifibrotic compared to patients not treated with antifibrotic

**Covariates include age, gender, smoking, FVC, DLCO, coronary artery disease, chronic obstructive pulmonary disease, pulmonary hypertension, and oxygen use

A sensitivity analysis including the 65 patients who were on antifibrotics for more than 0 but less than 180 days found similar results regarding clinical outcomes with the exception of the baseline USCD shortness of breath score, which was no longer significantly different between the two groups (Supplemental Tables 1–3).

## Discussion

In this study of the largest collection of tertiary ILD centers in the United States, we found that 63% of newly diagnosed IPF patients were treated continuously with antifibrotics for 180 days. Antifibrotic use was associated with younger age, less tobacco use, significantly lower mortality, and a numerically but not significantly lower hazard of transplant-free survival. Contrary to our hypothesis, we found an increased hazard of respiratory hospitalization and no statistically significant difference in time to FVC decline between treatment groups. We also found that patients treated continuously with antifibrotics had fewer baseline respiratory symptoms compared to those not treated.

Despite this cohort’s creation nearly two years after FDA approval of both pirfenidone and nintedanib for IPF, antifibrotic use was not universal. Of the four hundred and fifty-four patients diagnosed with IPF within a year of enrollment into the registry, two hundred and eighty-eight (63%) patients diagnosed with IPF in the past year were subsequently treated continuously with antifibrotics for 180 days, and 101 (22%) of newly diagnosed patients never received antifibrotic therapy during the study follow-up. Overall antifibrotic uptake in our study was higher than described in a recent large study by Kaul et al. of veterans with IPF (17%) and another study by Dempsey et al. investigating an insurance claims cohort (26%), neither of which restricted study patients to those diagnosed within the past year [12, 13]. This difference becomes even more apparent when considering both the Kaul and Dempsey cohorts only required a single prescription fill for either medication in comparison with our more stringent 180 continuous days of use criterion. The centers contained within PFF-PR are majority tertiary care ILD sites with familiarity in prescribing these medications, which could explain the higher uptake; the similar prescription rate in a recent analysis of the IPF-PRO database supports this conclusion [6]. Regardless, even within tertiary ILD centers, there remains a significant proportion of patients who are not receiving antifibrotics for six months or more.

Unsurprisingly, our study found that patients treated with antifibrotics were younger than those not treated and had a lower rate of ever smoking tobacco. Both of these differences likely reflect hesitancy among providers to prescribe antifibrotics to older smokers, either due to fear of side effects or delay in prescription until other interventions, such as tobacco cessation or assessment and/or treatment for concomitant COPD, is complete. While antifibrotics were associated with a statistically significant reduction in unadjusted mortality in our study, there was not a significant difference in transplant-free survival or measures of longitudinal pulmonary function between treated and untreated groups. These findings contrast not only with some RCTs demonstrating a reduction in rate of FVC decline in antifibrotics but also multiple retrospective cohort studies and a systematic review and meta-analysis finding a reduction in mortality with antifibrotics [4, 5, 7, 8, 14]. However, a recent retrospective analysis of the IPF-PRO database found similar results to our study, and another study found a survival benefit of antifibrotics only within the first two years of prescription [6, 15]. Thus, perhaps the longer follow-up time afforded by these multicenter, real-world cohorts could explain these discrepancies.

An unanticipated finding was an increase in hazard of respiratory hospitalization in patients treated with antifibrotics. Hospitalization and subsequent readmission are common outcomes in ILD patients [11]. Our findings diverge from a pooled analysis of RCTs showing a decrease in respiratory hospitalization with antifibrotics as well as a systematic review that found a reduction in risk of IPF exacerbation with treatment [14, 16]. However, the multicenter IPF-PRO analysis found a similar increase in hospitalization hazard and posited it was secondary to a likely combination of unmeasured confounding between treated and untreated groups, a potentially unintended result of longer survival of patients on antifibrotics, and the closer monitoring within the healthcare system that can occur with patients on antifibrotics. While an increase in general healthcare utilization has not been clearly associated with antifibrotic use compared to those not on antifibrotics, one study comparing nintedanib to pirfenidone did find higher global healthcare costs for patients on nintedanib [17].

Interestingly, patients on antifibrotic treatment had fewer symptoms at baseline compared to untreated patients. While this difference reflects a pre-treatment imbalance between groups, it contrasts with findings from IPF-PRO demonstrating worse quality of life scores in patients being treated [18]. As no clear relationship between antifibrotics and alleviation of symptom burden has been seen previously, this finding underscores that more study is needed regarding the relationship between symptom burden, antifibrotic use, and disease progression in IPF.

Strengths of this multicenter study include the real-world nature of patient enrollment as opposed to stringent RCT exclusion criteria, the rigorous methodology used to ensure continuous usage of antifibrotics to improve validity, and length of follow-up time. Despite attempts to limit immortal time bias and misclassification bias, an inherent limitation of this smaller, retrospective study is that these biases are not able to be completely ameliorated. While antifibrotics are indicated for patients with IPF regardless of disease severity, bias by indication could have resulted in the treatment of patients with more clinical risk factors for deterioration. This phenomenon could partially explain the higher rates of respiratory hospitalization. As the point estimates for transplant-free survival and overall survival favored the treatment group but were not statistically significant, statistical power and a corresponding underrecognition of a clinically significant effect could be possible.

In summary, this study of the multicenter PFF-PR demonstrated that 63% of patients with newly diagnosed IPF reported continuous antifibrotic usage for six months or more. Antifibrotic usage was not associated with improved transplant-free survival or longitudinal pulmonary function but was associated with an increased hazard of respiratory hospitalization. Future studies should further investigate the role of antifibrotic therapy as it relates to patient-centered outcomes, such as respiratory hospitalization and quality of life, in addition to studying real-world outcomes of antifibrotics on progressive pulmonary fibrosis outside of the IPF paradigm.

### Electronic supplementary material

Below is the link to the electronic supplementary material.

Supplementary Material 1

## Data Availability

Data will be provided by the authors on request.

## Abbreviations

CAD: Coronary artery disease
COPD: Chronic obstructive pulmonary disease
FDA: Food and Drug Administration
FVC: Forced vital capacity
ILD: Interstitial lung disease
IPF: Idiopathic pulmonary fibrosis
PFF-PR: Pulmonary fibrosis foundation patient registry
US: United States

## References

[1] Zheng Q, Cox IA, Campbell JA, et al. Mortality and survival in idiopathic pulmonary fibrosis: a systematic review and meta-analysis. ERJ Open Res Published Online January. 2022;1. 10.1183/23120541.00591-2021. 10.1183/23120541.00591-2021 PMC8918939 35295232

[2] Ruwanpura SM, Thomas BJ, Bardin PG (2020). Pirfenidone: Molecular mechanisms and potential clinical applications in Lung Disease. Am J Respir Cell Mol Biol.

[3] Wollin L, Wex E, Pautsch A (2015). Mode of action of nintedanib in the treatment of idiopathic pulmonary fibrosis. Eur Respir J.

[4] King TE, Bradford WZ, Castro-Bernardini S (2014). A phase 3 trial of pirfenidone in patients with idiopathic pulmonary fibrosis. N Engl J Med.

[5] Richeldi L, du Bois RM, Raghu G (2014). Efficacy and safety of nintedanib in idiopathic pulmonary fibrosis. N Engl J Med.

[6] de Andrade JA, Neely ML, Hellkamp AS (2023). Effect of Antifibrotic Therapy on survival in patients with idiopathic pulmonary fibrosis. Clin Ther.

[7] Kang J, Han M, Song JW (2020). Antifibrotic treatment improves clinical outcomes in patients with idiopathic pulmonary fibrosis: a propensity score matching analysis. Sci Rep.

[8] Behr J, Prasse A, Wirtz H, et al. Survival and course of lung function in the presence or absence of antifibrotic treatment in patients with idiopathic pulmonary fibrosis: long-term results of the INSIGHTS-IPF registry. Eur Respir J. 2020;56(2). 10.1183/13993003.02279-2019. 10.1183/13993003.02279-2019 32381492

[9] Wang BR, Edwards R, Freiheit EA (2020). The Pulmonary Fibrosis Foundation Patient Registry. Rationale, design, and methods. Ann Am Thorac Soc.

[10] King CS, Ignacio RV, Khangoora V, et al. Hospitalization rates in interstitial lung disease: an analysis of the pulmonary fibrosis foundation registry. Am J Respir Crit Care Med Published Online January. 2024;18. 10.1164/rccm.202309-1708OC. 10.1164/rccm.202309-1708OC 38236191

[11] Lee CT, Selvan K, Adegunsoye A (2024). Risk factors for Hospital Readmission in patients with interstitial lung disease. Respir Care. Published Online January.

[12] Kaul B, Lee JS, Petersen LA et al. Disparities in antifibrotic medication utilization among veterans with idiopathic pulmonary fibrosis. Chest. Published online February 18, 2023:S0012-3692(23)00273-8. 10.1016/j.chest.2023.02.027. 10.1016/j.chest.2023.02.027 PMC10410245 36801465

[13] Dempsey TM, Payne S, Sangaralingham L, Yao X, Shah ND, Limper AH (2021). Adoption of the Antifibrotic Medications Pirfenidone and Nintedanib for patients with idiopathic pulmonary fibrosis. Ann Am Thorac Soc.

[14] Petnak T, Lertjitbanjong P, Thongprayoon C, Moua T (2021). Impact of Antifibrotic Therapy on Mortality and Acute Exacerbation in Idiopathic Pulmonary Fibrosis: a systematic review and Meta-analysis. Chest.

[15] Dempsey TM, Sangaralingham LR, Yao X, Sanghavi D, Shah ND, Limper AH (2019). Clinical effectiveness of Antifibrotic Medications for idiopathic pulmonary fibrosis. Am J Respir Crit Care Med.

[16] Ley B, Swigris J, Day B, mo (2017). Pirfenidone reduces respiratory-related hospitalizations in idiopathic pulmonary fibrosis. Am J Respir Crit Care Med.

[17] Cottin V, Spagnolo P, Bonniaud P (2023). Healthcare resource use and associated costs in patients receiving pirfenidone or nintedanib for idiopathic pulmonary fibrosis. Respir Med Res.

[18] Salisbury ML, Conoscenti CS, Culver DA (2020). Antifibrotic Drug Use in patients with idiopathic pulmonary fibrosis. Data from the IPF-PRO Registry. Ann Am Thorac Soc.

