# Supplementary material for: The impact of antifibrotic use on long-term clinical outcomes in the pulmonary fibrosis foundation registry
Source: Respir Res. 2024 Jun 21;25:255. doi: 10.1186/s12931-024-02883-2 (PMC11193272; doi:10.1186/s12931-024-02883-2)
Supplement: Supplementary file 1 — Supplementary Material 1 [file 12931_2024_2883_MOESM1_ESM.docx]

**Supplemental Table 1.** Patient Characteristics (including patients on antifibrotic less than 180 days)

|  | Total Cohort (n=454) | Antifibrotic  (n=353) | No Antifibrotic  (n=101) | P-value* |
| --- | --- | --- | --- | --- |
| Age, mean (SD), years | 70.8(8.0)  (n=454) | 70.3(7.5)  (n=353) | 72.6(9.5)  (n=101) | 0.02 |
| Male, n (%) | 368(81.1) (n=454) | 285(80.7)  (n=353) | 83(82.2)  (n=101) | 0.74 |
| Race  White, n (%)  Black, n (%)  Asian, n (%) | 420(95.9)  4(0.9)  14(3.2)  (n=438) | 330(96.5)  3(0.9)  9(2.6)  (n=342) | 90(93.8)  1(1.0)  5(5.2)  (n=96) | 0.41 |
| Hispanic, n (%) | 21(4.8)  (n=435) | 16(4.7)  (n=340) | 5(5.3)  (n=95) | 0.79 |
| Body mass index, mean (SD) | 29.2(5.1)  (n=419) | 29.2(5.0)  (n=328) | 29.2(5.6)  (n=91) | 0.97 |
| Days since diagnosis, mean (SD) | 136.5(118.7)  (n=454) | 144.1(118.8)  (n=353) | 109.9(115.1)  (n=101) | 0.01 |
| Region  West, n (%)  Midwest, n (%)  South, n (%)  Northeast, n (%) | 101(22.3)  73(16.1)  187(41.2)  93(20.5)  (n=454) | 81(23.0)  52(14.7)  147(41.6)  73(20.7)  (n=353) | 20(19.8)  21(20.8)  40(39.6)  20(19.8)  (n=101) | 0.52 |
| Insurance  Private, n (%)  Medicare, n (%)  Others, n (%) | 243(53.5)  181(39.9)  30(6.6)  (n=454) | 190(53.8)  138(39.1)  25(7.1)  (n=353) | 53(52.5)  43(42.6)  5(5.0)  (n=101) | 0.67 |
| History of smoking tobacco, n (%) | 295(65.0)  (n=454) | 222(62.9)  (n=353) | 73(72.3)  (n=101) | 0.08 |
| No. of medical comorbidities, mean (SD) | 2.0(1.5)  (n=454) | 2.0(1.5)  (n=353) | 2.1(1.8)  (n=101) | 0.62 |
| Medical comorbidities  GERD, n (%)  OSA, n (%)  Arrhythmia, n (%)  CAD, n (%)  CHF, n (%)  COPD, n (%)  Cancer, n (%)  Depression, n (%)  Diabetes, n (%)  Cirrhosis, n (%)  Obesity, n (%)  PAH, n (%) | 49(10.8)  98(21.6)  54(11.9)  112(24.7)  18(4.0)  38(8.4)  77(17.0)  60(13.2)  84(18.5)  2(0.4)  75(16.5)  14(3.1)  (n=454) | 34(9.6)  74(21.0)  41(11.6)  87(24.7)  13(3.7)  28(7.9)  55(15.6)  47(13.3)  70(19.8)  1(0.3)  59(16.7)  10(2.8)  (n=288) | 15(14.9)  24(23.8)  13(12.9)  25(24.8)  5(5.0)  10(9.9)  22(21.8)  13(12.9)  14(13.9)  1(1.0)  16(15.8)  4(4.0)  (n=101) | 0.14  0.55  0.73  0.98  0.57  0.53  0.14  0.91  0.17  0.40  0.84  0.52 |
| Family history of ILD  Yes, n (%)  No, n (%)  Unknown, n (%) | 74(16.3)  325(71.6)  55(12.1)  (n=454) | 59(16.7)  252(71.4)  52(11.9)  (n=353) | 15(14.9)  73(72.3)  13(12.9)  (n=101) | 0.89 |
| SD=standard deviation; GERD=gastro-esophageal reflux disease, OSA=obstructive sleep apnea, CAD=coronary artery disease, CHF=congestive heart failure, COPD=chronic obstructive pulmonary disease, PAH=pulmonary hypertension, ILD=interstitial lung disease | | | | |

**Supplemental Table 2**. Pulmonary Function, Oxygen Use, and Patient-Reported Outcomes by Antifibrotic Use (including patients on antifibrotic less than 180 days)

|  | Total Cohort (n=454) | Antifibrotic  (n=353) | No Antifibrotic  (n=101) | P-value |
| --- | --- | --- | --- | --- |
| FVC % predicted, baseline, mean (SD) | 69.4(16.8)  (n=368) | 69.1(16.3)  (n=289) | 70.6(18.7)  (n=79) | 0.49 |
| DLCO uncorrected % predicted, baseline, mean (SD) | 44.0(15.0)  (n=323) | 43.6(14.7)  (n=256) | 45.7(16.2)  (n=67) | 0.29 |
| Gender-Age-Physiology score | 4.3(1.3)  (n=323) | 4.3(1.3)  (n=256) | 4.2(1.3)  (n=67) | 0.92 |
| Supplemental oxygen use, n (%) | 176(39.3)  (n=448) | 143(41.0)  (n=349) | 33(33.3)  (n=99) | 0.17 |
| Fatigue Severity Scale score, mean (SD) | 4.0(1.8)  (n=435) | 3.9(1.7)  (n=339) | 4.3(1.9)  (n=96) | 0.04 |
| Leicester Cough Questionnaire score, mean (SD) | 16.9(3.5)  (n=435) | 17.0(3.4)  (n=340) | 16.3(3.9)  (n=95) | 0.09 |
| Rand SF-6D Health-related Quality of Life score, mean (SD) | 0.7(0.1)  (n=436) | 0.7(0.1)  (n=340) | 0.7(0.1)  (n=96) | 0.22 |
| UCSD Shortness of Breath-score, mean (SD) | 35.3(25.3)  (n=432) | 34.4(24.4)  (n=338) | 38.4(28.1)  (n=94) | 0.18 |
| Death (all-cause), n (%) | 148(32.6)  (n=454) | 113(32.0)  (n=353) | 35(34.7)  (n=101) | 0.62 |
| Respiratory hospital visit, n (%) | 122(26.9)  (n=454) | 110(31.2)  (n=353) | 12(11.9)  (n=101) | 0.0001 |
| FVC=forced vital capacity; DLCO=diffusing capacity of carbon monoxide; UCSD=University of California San Diego | | | | |

**Supplemental Table 3.** Hazard Ratios of Death, Lung Transplant, and Hospitalization by Antifibrotic Use (including patients on antifibrotic less than 180 days)

| **Outcome** | **Unadjusted HR*** | | **Adjusted HR**** | |
| --- | --- | --- | --- | --- |
|  | Hazard ratio (95% CI) | P-value | Hazard ratio (95% CI) | P-value |
| Death/Transplant | 0.987 (0.706, 1.380) | 0.94 | 0.971 (0.657, 1.434) | 0.88 |
| Death | 0.792 (0.541, 1.158) | 0.23 | 0.871 (0.556, 1.365) | 0.55 |
| Respiratory hospitalization | 2.476 (1.364, 4.495) | 0.003 | 2.382 (1.184, 4.788) | 0.01 |
| FVC 10% decline^$^ | 1.500 (0.900,2.498) | 0.12 | 1.519 (0.905, 2.548) | 0.11 |
| FVC 5% decline^%^ | 1.143 (0.774,1.687) | 0.50 | 1.101 (0.740,1.641) | 0.63 |
| *Hazard ratio-hazard of outcome in patients treated with antifibrotic compared to patients not treated with antifibrotic  **Covariates include age, gender, smoking, FVC, DLCO, coronary artery disease, chronic obstructive pulmonary disease, pulmonary hypertension, and oxygen use | | | | |
